# Consequences of Transport Conditions on the Welfare of Slaughter Pigs with Different Health Status and RYR-1 Genotype

**DOI:** 10.3390/ani14020191

**Published:** 2024-01-06

**Authors:** Nikola Čobanović, Sara Čalović, Branko Suvajdžić, Nevena Grković, Sanja Dj Stanković, Milena Radaković, Kristina Spariosu, Nedjeljko Karabasil

**Affiliations:** 1 Department of Food Hygiene and Technology, Faculty of Veterinary Medicine, University of Belgrade, Bulevar oslobodjenja 18, 11000 Belgrade, Serbia; brankos@vet.bg.ac.rs (B.S.); nevena.ilic@vet.bg.ac.rs (N.G.); nedja@vet.bg.ac.rs (N.K.); 2 Department of Animal Breeding, Faculty of Veterinary Medicine, University of Belgrade, Bulevar oslobodjenja 18, 11000 Belgrade, Serbia; sara_kovachevic@yahoo.com; 3 Center for Medical Biochemistry, University Clinical Center of Serbia, 11000 Belgrade, Serbia; sanjast2013@gmail.com; 4 Faculty of Medical Sciences, University of Kragujevac, 34000 Kragujevac, Serbia; 5 Department of Pathophysiology, Faculty of Veterinary Medicine, University of Belgrade, Bulevar oslobođenja 18, 11000 Belgrade, Serbia; mradakovic@vet.bg.ac.rs (M.R.); kristina@vet.bg.ac.rs (K.S.)

**Keywords:** acute-phase proteins, behaviour, oxidative stress biomarkers, physiometabolic blood profile, short transport distance, subclinical pathological lesions, welfare

## Abstract

**Simple Summary:**

During transportation, slaughter pigs are subjected to a number of different stressors that could compromise their welfare, health and performance, as well as industry’s profitability. Thus, it is of paramount importance to verify the slaughter pigs’ condition throughout the pork supply chain to ensure their well-being and health and to obtain high-quality carcasses and meat. To date, there is no information on the response of slaughter pigs with different health status and RYR-1 genotype to transport procedures. Based on this, the aim of this study was to assess the influence of transport conditions on welfare of slaughter pigs with different health status and RYR-1 genotype. The most compromised welfare was recorded in subclinically diseased stress-carrier pigs exposed to short transportation (<30 min) and high loading density (~235 kg/m^2^), while under the same conditions the welfare of healthy stress-resistant pigs was not compromised. Accordingly, it can be concluded that stress-carrier pigs with subclinical pathological lesions should not be considered fit for transportation, indicating that the health status and genotype are the key factors for optimising pig welfare.

**Abstract:**

This study assessed the influence of transport conditions on welfare indicators of slaughter pigs with different health status and RYR-1 genotype. The group of pigs, predominantly consisting of Nn (56.67%) and subclinically diseased (60.00%) individuals, that were exposed to short transportation (<30 min) at high loading density (~235 kg/m^2^) had the highest slipping (*p* < 0.0001), falling (*p* = 0.0009), turning back (*p* < 0.0001), reluctance to move (*p* < 0.0001), panting (*p* < 0.0001) and shivering (*p* < 0.0001) frequencies at unloading. Subclinically diseased Nn pigs subjected to short transportation (<30 min) and high loading density (~235 kg/m^2^) had the highest lactate (*p* < 0.0001 and *p* < 0.0001), glucose (*p* = 0.0450 and *p* = 0.0002), CK (*p* < 0.0001 and *p* = 0.0010), LDH (*p* < 0.0001 and *p* = 0.0484), AST (*p* = 0.0208 and *p* = 0.0170), ALT (*p* = 0.0500 and *p* = 0.00081), ceruloplasmin (*p* = 0.0334 and *p* < 0.0001) and MDA (*p* = 0.0048 and *p* < 0.0001) concentrations, but the lowest sodium (*p* < 0.0001 and *p* < 0.0001), chloride (*p* = 0.0001 and *p* = 0.0432), albumin (*p* < 0.0090 and *p* < 0.0001), PON-1 (*p* = 0.0122 and *p* = 0.0500) and GSH (*p* = 0.0042 and *p* = 0.0340) levels, respectively. In the group consisting of of stress-resistant (100%) and predominantly healthy (60.00%) pigs subjected to short transportation (<30 min) at high loading density (~235 kg/m^2^), none of the individuals showed irregular behavioural reactions during unloading. Healthy NN pigs that underwent short transportation (<30 min) at high loading density (~235 kg/m^2^) had the lowest lactate (*p* < 0.0001 and *p* < 0.0001), glucose (*p* = 0.0450 and *p* = 0.0002), CK (*p* < 0.0001 and *p* = 0.0010), LDH (*p* < 0.0001 and *p* = 0.0484) and ceruloplasmin (*p* = 0.0334 and *p* < 0.0001) levels, but the highest sodium (*p* < 0.0001 and *p* < 0.0001) and chloride (*p* = 0.0001 and *p* = 0.0432) concentrations, respectively. In conclusion, the most compromised welfare was recorded in subclinically diseased Nn pigs exposed to short transportation (<30 min) and high loading density (~235 kg/m^2^), while under the same conditions, the welfare of healthy NN pigs was not compromised. Therefore, stress-carrier pigs with subclinical pathological lesions should not be considered fit for transportation, indicating that the health status and genotype are the key factors for optimising pig welfare.

## 1. Introduction

Animal well-being during transportation is of the interest to the public, scientific community, consumers, primary producers and meat industry, since a large number of livestock intended for farming and slaughter are transported every day throughout the world to meet market needs [1,2,3]. Even under favourable transport conditions, livestock are subjected to a myriad of potential stressors that could compromise their welfare, health and performance, as well as the industry’s profitability [4]. Hence, it is of paramount importance to verify the livestock condition throughout the supply chain to ensure their well-being and health and to obtain high-quality carcasses and meat [5]. Among the events during the pre-slaughter period of fattening pigs, transportation time, loading density and severe climate conditions are considered to be the major factors that can severely deteriorate the health and overall welfare of animals and, thus, have negative effects on carcass yield and pork quality [5,6,7,8,9].

Each stage of transportation—loading, the journey itself and unloading—expose pigs to various stressful factors that could be psychological (separation and/or mixing of different species or animals from different origins, inadequate loading density, fights, human–animal contact, exposure to novel surroundings) or physical (withholding of water and feed for long periods, noise, vibration, acceleration and associated fatigue, skin bruises, lack of thermal comfort) [4,5]. Consequently, transportation could cause stress to pigs in different degrees, varying from mild discomfort to extreme distress (and even death), which induce behavioural, haematological, physiological and neurohormonal changes [5]. Any period of transportation, especially under poor conditions, was associated with irregular behavioural reactions, hypercortisolemia, hyperglycaemia, alterations in red and white blood cell parameters and electrolytes, increases in concentrations of creatine kinase (CK), lactate dehydrogenase (LDH), aspartate amino transferase (AST), alanine amino transferase (ALT), oxidative stress products and acute phase proteins [10,11,12,13,14,15,16]. Moreover, when inadequate transport conditions are combined with a genetic mutation of pigs that carry the recessive ryanodine receptor (RYR-1) gene (n), the aforementioned stressful factors can trigger more severe metabolic alterations known as porcine stress syndrome [5,17].

Additionally, long transportation and longer waiting periods in the lairage pens before slaughter could increase susceptibility to infection, triggering the onset of health problems in different food animal species, such as transport pneumonia and gastrointestinal diseases in horses [18,19], bovine respiratory disease in cattle [4,20], heart diseases in broilers [21,22], pneumonia and salmonellosis in sheep [23] and acute pleuropneumonia in pigs [24,25]. However, to the best of the authors’ knowledge, there is no study reporting welfare outcomes after short transportation of subclinically diseased animals. Despite the fact that, in most countries, the transportation of food animals showing disease signs is prohibited, the problems arising from subclinically diseased individuals and difficulties of detection remain unresolved [26]. This issue is gaining particular importance as most loading of food animals for transportation is carried out by drivers and farmers (without a veterinarian present), who are not competent to correctly identify diseased individuals [26]. In addition to a considerable increase in stress susceptibility and impaired meat quality, pigs containing the mutant n allele (nn and Nn genotypes) have increased susceptibility to diseases and higher predisposition for the occurrence of subclinical diseases [27,28]. Overall, the complex interactions between transportation stressors, pre-existing health conditions and genetic differences complicate the identification of cause–effect relationships and the measurement of the magnitude of their impact on pig welfare. Therefore, understanding trends in the welfare parameters of slaughter pigs with different health status and RYR-1 genotype is of primary importance for the development, adaptation and implementation of preventive measures, which in turn would mitigate the negative effects of transportation and improve pig welfare standards. In light of this brief review of the related scientific literature, the objective of this study was to determine the influence of different transport conditions on antemortem and postmortem welfare indicators of slaughter pigs with different health status and RYR-1 genotype.

## 2. Materials and Methods

### 2.1. Ethical Statement

The investigation was conducted in line with the European legal requirements for the protection of pigs on farms (Directive 2008/120/EC [29]), during transportation (Council Regulation-ECN 1/2005 [30]) and at slaughter (Council Regulation -EC- N 1099/2009 [31]). The slaughter pigs were raised on two commercial farms and slaughtered for human meat consumption at the commercial abattoir in compliance with aforementioned legislation. Pigs were not exposed to any experimental invasive procedure in vivo (blood samples were taken during slaughtering, while skin bruises were evaluated on the carcasses in the chilling chamber). For these reasons, this experiment did not fall within the field of application of Directive 2010/63/EU [32] on the protection of animals used for scientific purposes and thus did not require a specific authorisation by the local animal welfare and ethical review body.

### 2.2. Experimental Animals, Pre-Slaughter Management and Slaughter Procedure

The experiment was performed during spring on 240 slaughter pigs (119 barrows and 121 gilts) referring to eight shipments (30 animals/shipment). Slaughter pigs were of the same genetics ([Yorkshire × Landrace] sows sired with Pietrain boars) with an average live weight of approximately 110 kg and were about 6 months old. Pigs originated from two commercial farrow-to-finish farms (each producing about 2000 fattening pigs per year) belonging to a single company and subjected to uniform and standardised rearing conditions. Both farms used all in–all out management by pen and housed 30 individuals in similar standard-sized pens (3.0 × 10.0 × 2.0 m) with a minimum of 1 m^2^ per pig. All pens had natural light conditions, the same type of floors, walls, doors and ceiling. The piglets were transferred to the finishing units at approximately 30 kg body weight and about 3 months old. During the growing-finishing period, the pigs had ad libitum access to the same standard commercial diets (formulated to meet National Research Council nutrient recommendations [33]) and water via an automatic feeder and three nipple drinkers in each pen. Pigs were inspected daily, and no health problems were recorded during the growing-finishing period. On both farms, the pigs were sent to slaughter after reaching the body weight of 110.00 ± 2.00 kg (180 days old). Prior to transportation, pigs were fasted for 16 h, but drinking water was always freely available during housing.

At the farms, the pigs were loaded (always in the back lorry compartment) in groups of six individuals by the same driver and the same farm loading crew following normal commercial Serbian procedures using sorting boards and, if necessary, electric prods. Loading time ranged from 35 to 82 min. Lorry departed from the farms immediately after loading. All transports were carried out using a smooth driving style with the same lorry and same lorry driver. Data collection was intentionally performed under commercial pre-slaughter conditions, and thus no instructions regarding handling of the slaughter pigs were provided to the lorry driver and the farm loading crew.

The distances from the Farm A and Farm B to a commercial abattoir was 215 km and 5 km, respectively. Pigs were transported from Farm A to the abattoir over more than three hours (on average 227.50 ± 14.43 min), while pigs were transported from Farm B to the abattoir in less than 30 min (on average 22.75 ± 1.71 min), depending on the farm distance, transport route and traffic jams. The short transportation time was chosen to represent the situation in which pigs experienced a typical amount of acute stress during the day of slaughter, while the long transportation time represented typical average journey times from the farm to the abattoir for slaughter pigs in Serbia. Depending on the body weight and number of slaughter pigs in the lorry, the loading density ranged from 198 to 236 kg/m^2^. Each farm of origin provided four shipments of pigs (120 pigs per farm). Pigs were slaughtered in April (four shipments) and May (four shipments). On sampling days, the ambient temperature ranged between 14.5 °C and 21.0 °C, the relative humidity fluctuated from 42.0% to 65.5%, while the temperature-humidity index varied from 59.22 to 66.13. A shipment was defined as “a group of pigs exposed to the same pre-slaughter treatment (originating from the same farm, transported at the same time on the same lorry and subjected to the same lairage and slaughter conditions)”. Each group of pigs was unloaded as soon as possible upon the arrival at the abattoir. Waiting time prior to unloading varied from 10 to 25 min. Unloading for each shipment was carried out by the same abattoir personnel using the lorry ramp (10° angle; no bedding on the floor was added to the ramp) and pigs were driven for 10 m to the lairage pens (0.65 m^2^/pig), where they rested for about one hour. Unloading time ranged from 10 to 65 min. During lairaging, pigs had ad libitum access to drinking water, but there was no access to food. Lairage temperature and relative humidity followed outside conditions. During antemortem inspection in lairage pens carried out by official veterinary inspectors, no clinical signs of disease were detected in any examined pig. The lorry driver was interviewed through the aid of structured questionnaires to collect data about farm of origin, daily temperature and relative humidity during loading, loading time, transportation time, distance from farm to abattoir, loading density, waiting time and unloading time. Also, information regarding the lairage time, lairage density and daily temperature and relative humidity (during unloading and lairaging) was obtained from abattoir staff. Temperature-humidity index was calculated based on the ambient temperature (AT; expressed as °C) and relative humidity (RH; expressed as fraction of a unit) at loading, unloading and at lairage by using the following formula [34]: THI = (1.8 × AT + 32) − [0.55 × (RH/100)] × [(1.8 × AT + 32) − 58]. The data regarding pre-slaughter conditions are summarised in Table 1. After lairaging, pigs were slaughtered during the morning hours in line with the standard industry-accepted practices in the same low-input commercial abattoir that operates from Monday to Saturday (07:00–15:00 h) and has a slaughtering capacity of 175 pigs/week.

### 2.3. Sample Size Determination

The sample size was calculated using G*Power software (Version 3.1.9.2, University Kiel, Germany) [35]. In the power analysis utilising a Chi-squared goodness-of-fit test (contingency tables), with input parameters including an effect size of 0.35, an α level of 0.05, a power of 0.95 and degrees of freedom (df) set at 7, the determined sample size (n) amounted to 179 pigs. For the power analysis employing an F-test ANOVA (fixed effects, omnibus and one-way), considering an effect size of 0.45, an α level of 0.05, a power of 0.95 and eight groups, the required sample size (n) totalled 120 pigs, with 15 pigs allocated to each group.

### 2.4. Antemortem Welfare Indicators

Antemortem welfare indicators of slaughter pigs were observed at unloading and in the lairage based on the Welfare Quality^®^ protocol [36], as outlined in Table 2. Behavioural and health measurements were monitored by two trained assessors and included the following: (i) slipping, falling, turning back, reluctance to move, panting, shivering, sick animals and dead animals during unloading; (ii) coughing, sneezing, panting, shivering, huddling and dead animals during lairaging.

### 2.5. Collection, Selection and Preparation of Blood Samples

At the beginning of exsanguination during slaughtering, samples consisted of 100 mL of trunk blood were taken from each pig into a plastic cup. To determine RYR1 genotype, blood was immediately transferred to vacutainers (2 mL) coated with EDTA. For determination of physiometabolic blood profile and oxidative stress biomarkers, blood was transferred to two types of tubes: the first tube was treated with potassium oxalate/sodium fluoride to inhibit further glycolysis, and the second tube was treated with EDTA for anti-coagulation. All types of tubes were inverted gently eight times immediately after collection. After collection, each blood sample was uniquely labelled with a number corresponding to the carcass kill/line number. For transportation to the laboratory, tubes containing EDTA were placed on ice packs (maintained at 2 ± 1 °C) within a cooler box. Upon prompt arrival at the laboratory, approximately 2 h post-sampling, the blood samples underwent centrifugation at 3000 rpm for 10 min. Plasma aliquots were then transferred into tubes, individually labelled, and promptly frozen at −80 °C until required for testing (approximately 30 days post-sampling).

### 2.6. RYR1 Genotype Determination

RYR1 genotype (NN—stress-resistant; Nn—stress carrier; nn—stress-susceptible) was determined using the PCR-RFLP (Polymerase Chain Reaction—Restriction Fragment Length Polymorphism) method based on Brenig and Brem [37], as outlined in Čobanović et al. [28]. From the results obtained through PCR-RFLP, genotype frequencies of the RYR-1 locus were calculated for the examined slaughter pigs.

### 2.7. Postmortem Welfare Indicators

#### 2.7.1. Physiometabolic Biomarker Quantification

The concentrations of lactate and glucose in the blood were measured within 10 min of exsanguination at the slaughterline using portable devices (blood glucose: Accu-chek^®^ Performa, Roche Diagnotics, Mannheim, Germany; blood lactate: Accutrend Plus, Roche Diagnostics, Roche, Mannheim, Germany) from the first tubes (treated with potassium oxalate/sodium fluoride). The concentrations of cortisol and adrenocorticotropic hormone (ACTH) in plasma were analysed using an automated analyser (Roche Cobas e601, Roche Diagnostics, Mannheim, Germany). The analytical sensitivities (lower detection limit) for ACTH and cortisol were 0.220 pmol/L and 0.500 nmol/L, respectively. The levels of potassium, sodium, chloride, calcium, CK, LDH, AST, ALT, haptoglobin, C-reactive protein (CRP) and albumin in plasma were assessed using an automated analyser (Architect c8000, Abbott, Wiesbaden, Germany). The concentration of ceruloplasmin was measured upon its p-phenylenediamine (PPD) oxidase activity according to Hussein et al. [38]. The absorbance of the purple-coloured product was read at 530 nm. Paraoxonase-1 (PON-1) activity in plasma aliquots was evaluated using 4-nitrophenil acetate as a substrate [39]. The rate of 4-nitrofenol produced was continuously monitored at 402 nm (ε 402 = 14,000 M^−1^ cm^−1^ at pH 7.4). Each assay was conducted in triplicate, and the final result was determined by averaging the values of the three measurements. For physiometabolic blood parameters, the intra-assay coefficients of variation were consistently below 10%.

#### 2.7.2. Oxidative Stress Biomarker Quantification

Malondialdehyde (MDA), as indicator of lipid peroxidation, was quantified in plasma aliquots by measuring the formation of thiobarbituric acid reactive substances (TBARS) as MDA equivalents [40]. The glutathione (GSH) levels in plasma aliquots were determined according to the modified Ellman’s method [41] with 5,5′-dithiobis-(2-nitrobenzoic) acid (DTNB). The absorbance was read at 412 nm. Each assay was conducted in triplicate, and the final result was determined by averaging the values of the three measurements. For oxidative stress biomarkers, the intra-assay coefficients of variation were consistently below 10%.

#### 2.7.3. Pluck Examination

Examination of the pluck of slaughtered pigs (heart, lung and liver set from each pig) was performed via visual inspection and manual palpation of the organs for macroscopically visible lesions of pneumonia, pleurisy, pericarditis and liver milk spots (indicative of the transhepatic migration of the *Ascaris suum* larvae) based on the Welfare Quality^®^ protocol [36]. A positive case for each pathological lesion was defined as a pig organ affected with any degree of the lesion (score 2) and a negative case when lesions were absent (score 0). The complete assessment of pathological lesions was performed by a single observer throughout the study to eliminate inter-observer variation.

#### 2.7.4. Carcass Bruise Evaluation

Carcass bruises were visually assessed on the left carcass side in the chilling chamber 45 min after slaughter using a visual scoring system based on Welfare Quality^®^ protocol [36]. Carcass bruises were assessed by a single observer throughout the study to eliminate inter-observer variation. The same observer recorded gender, herd identification number and kill number. Approximately one minute per pig was needed to record bruises on the carcass. Also, carcass bruises were classified as handling-type, fighting-type and mounting-type by visual assessment of shape and size to recognise their origin [42]. Only recent/fresh carcass bruises were recorded. The bruises were considered to be fresh if it was bright red in colour or with apparently recent and intact scabs. Carcass bruises smaller than two centimetres, bruised injection sites and reddening lesions on the left hind leg that looked like bruises more likely caused after stunning by the tightening of the shackle chain were not registered.

#### 2.7.5. Rigor Mortis Intensity Determination

Rigor mortis intensity was determined by objective and subjective method. Objective rigor mortis intensity was determined on the left carcass side 45 min postmortem by measuring the degree of angle between the body axis and foreleg according to Čobanović et al.’s method [43]. Subjective assessments of the rigor development were made 45 min postmortem on the *Musculus semimembranosus* in the split carcass using a three-point scale according to Čobanović et al. [43].

### 2.8. Statistical Analysis

Data were entered into Microsoft Excel 2016 (Microsoft Corporation, Redmond, WA, USA) and statistical analysis of the results was conducted with SPSS software (Version 23.0, IBM Corporation, Armonk, NY, USA) [44]. The linearity, normality of residuals (Shapiro–Wilk and Kolmogorov–Smirnov test), outliers and homogeneity of variance (Levene’s test) of the dependent variables were determined before statistical analysis, and data successfully passed all tests. According to RYR1 genotype, the pigs were allocated to two groups: (i) Nn pigs: stress-carriers (*n =* 87) and (ii) NN pigs: stress-resistant pigs (*n =* 153), while pigs were classified into two groups based on the health status: (i) healthy pigs: slaughtered pigs without clinical signs of disease at antemortem examination and pathological lesions in lungs, liver and heart at postmortem examination (*n =* 92); (ii) subclinically diseased pigs: slaughtered pigs without clinical signs of disease at antemortem examination but with any sign of pathological lesions in lungs, liver and heart at postmortem examination (*n =* 148). Based on loading density, pigs were classified in two groups using the threshold for optimal loading density (0.425 m^2^/100 kg pig) recommended by European Union (EU) regulations [30]: (i) high loading density: space allowance in the lorry within or slightly lower than recommended density of 235 kg/m^2^ (*n =* 120); low loading density: the space allowance in the lorry higher than 235 kg/m^2^ (*n =* 120). In addition, animals were divided into two groups for transportation time: (i) short transportation (<30 min) (*n =* 120); and (ii) long transportation (>3.5 h) (*n =* 120).

Shipment and individual pig were considered an experimental unit, depending on the statistical test. Potassium and calcium were excluded from all statistical tests as their concentrations were below the limits of detection. In addition, percentage of sick and dead animals at unloading, as well as frequency of panting, shivering, huddling, coughing, sneezing and dead animals at lairage were not included in the statistical analysis, since none of the pigs from any shipment showed this kind of irregular behaviour. Statistical significance was accepted at *p* < 0.05, while tendencies were accepted at 0.05 < *p* < 0.10. The Chi-squared test was used to determine differences between shipments for antemortem welfare indicators. The differences between shipments were not significant for postmortem welfare indicators and, therefore, were removed from the model. General Linear Mixed Model (GLMM) analysis, considering transportation time, loading density, gender, RYR-1 genotype and health status (2 × 2 × 2 × 2 × 2) as a fixed effects and shipment as a random effect, was used to determine their influence on postmortem indicators. All two-, three-, four- and five-way interactions between fixed effects were tested and removed from the model if *p* > 0.05. The random (shipment) effect was not significant (*p* > 0.05) for any of the examined postmortem welfare indicators. Three-way interactions were significant (loading density × RYR-1 × health status and transportation time × RYR-1 × health status) and retained in the final model for describing significant effects. For all examined postmortem welfare parameters (except for carcass bruise severity and type), the inter-group comparisons were appraised via one-way Analysis of Variance (ANOVA) followed by Tukey’s multiple comparison tests (difference considered significant if *p* < 0.05). The data were described as means with standard errors of the means (SEM). Significant differences for carcass bruise severity and type between groups were evaluated using the Chi-squared test. It was not possible to conduct GLMM analysis at the individual level for antemortem welfare indicators because assessors were not able to identify each individual pig during examination.

## 3. Results

### 3.1. Antemortem Welfare Indicators Based on Transport Conditions, RYR-1 Genotype and Health Status of Slaughter Pigs

Differences in frequency of RYR-1 genotypes and health status of slaughter pigs in relation to transport conditions are shown in Table 3, while differences in antemortem welfare indicators at the shipment level are depicted in Table 4. The group of slaughter pigs (Shipment 8), predominantly consisting of stress-carrier (Nn genotype, 56.67%) and subclinically diseased (60.00%) individuals, that were exposed to short transportation (<30 min) at high loading density (~235 kg/m^2^) had the highest percentage of slipping (*p* < 0.0001), falling (*p* = 0.0009), turning back (*p* < 0.0001), reluctance to move (*p* < 0.0001), panting (*p* < 0.0001) and shivering (*p* < 0.0001) at unloading (Table 3 and Table 4). In contrast, the group of slaughter pigs (Shipment 3), consisting of stress-resistant (NN genotype, 100%) and predominantly healthy (60.00%) individuals, that were subjected to long transportation (>3.5 h) at high loading density (~235 kg/m^2^) had the lowest (*p* < 0.0001) frequency of slipping at unloading (Table 3 and Table 4). In addition, none of the individuals from aforementioned group showed any sign of other irregular behavioural reactions during unloading and at lairage (Table 3 and Table 4).

### 3.2. Influence of Loading Density on the Physiometabolic Blood Profile of Slaughter Pigs with Different Health Status and RYR-1 Genotype

Differences in pathological lesions in slaughter pigs in relation to loading density, RYR-1 genotype and health status are presented in Table 5. The influence of loading density on the physiometabolic blood profile of slaughter pigs with different health status and RYR-1 genotype is displayed in Table 6. Subclinically diseased Nn pigs subjected to high loading density (~235 kg/m^2^) during transportation had the highest concentrations of lactate (*p* < 0.0001), glucose (*p* = 0.0450), CK (*p* < 0.0001), LDH (*p* < 0.0001), AST (*p* = 0.0208), ALT (*p* = 0.0500), ceruloplasmin (*p* = 0.0334) and MDA (*p* = 0.0048), but the lowest levels of sodium (*p* < 0.0001), chloride (*p* = 0.0001), albumin (*p* < 0.0001), PON-1 (*p* = 0.0122) and GSH (*p* = 0.0042). In contrast, healthy NN pigs exposed to high loading density (~235 kg/m^2^) during transportation had the lowest levels of lactate (*p* < 0.0001), glucose (*p* = 0.0450), CK (*p* < 0.0001), LDH (*p* < 0.0001), ceruloplasmin (*p* = 0.0334) and MDA (*p* = 0.0048), but the highest sodium (*p* < 0.0001), chloride (*p* = 0.0001), albumin (*p* < 0.0001), PON-1 (*p* = 0.0122) and GSH (*p* = 0.0042) concentrations (Table 6).

The influence of loading density on the rigor mortis and carcass bruises of slaughter pigs with different health status and RYR-1 genotype is shown in Table 7. Subclinically diseased Nn pigs subjected to high loading density (~235 kg/m^2^) during transportation had the most developed rigor mortis (the smallest foreleg angle) (*p* = 0.0018) and the highest percentage of severe carcass bruises (*p* < 0.0001) and handling-type carcass bruises (*p* < 0.0001). In contrast, healthy NN pigs exposed to high loading density (~235 kg/m^2^) during transportation had the least developed rigor mortis (the biggest foreleg angle) (*p* = 0.0018), the highest percentage of carcasses free of bruises (*p* < 0.0001) and the lowest percentage of handling-type carcass bruises (*p* < 0.0001) (Table 7).

### 3.3. Influence of Transportation Time on the Physiometabolic Blood Profile of Slaughter Pigs with Different Health Status and RYR-1 Genotype

Differences in pathological lesions in slaughter pigs in relation to transportation time, RYR-1 genotype and health status is presented in Table 8. The influence of transportation time on the physiometabolic blood profile of slaughter pigs with different health status and RYR-1 genotype is displayed in Table 9. Subclinically diseased Nn pigs subjected to short transportation (<30 min) had the highest concentrations of lactate (*p* < 0.0001), glucose (*p* = 0.0002), CK (*p* = 0.0010), LDH (*p* = 0.0484), AST (*p* = 0.0170), ALT (*p* = 0.0081), ceruloplasmin (*p* < 0.0001) and MDA (*p* < 0.0001), but the lowest levels of sodium (*p* < 0.0001), chloride (*p* = 0.0432), albumin (*p* = 0.0090), PON-1 (*p* = 0.0500) and GSH (*p* = 0.0340). In contrast, healthy NN pigs that underwent short transportation (<30 min) had the lowest levels of lactate (*p* < 0.0001), glucose (*p* = 0.0002), CK (*p* = 0.0010), LDH (*p* = 0.0484) and ceruloplasmin (*p* < 0.0001), but the highest sodium (*p* < 0.0001) and chloride (*p* = 0.0432) concentrations (Table 9).

The influence of transportation time on the rigor mortis and carcass bruises of slaughter pigs with different health status and RYR-1 genotype is shown in Table 10. Subclinically diseased Nn pigs that underwent short transportation (<30 min) had the most developed rigor mortis (the smallest foreleg angle) (*p* < 0.0001) and the highest percentage of severe carcass bruises (*p* < 0.0001) and handling-type carcass bruises (*p* < 0.0001). In contrast, healthy NN pigs subjected to short transportation (<30 min) had the least developed rigor mortis (the biggest foreleg angle) (*p* < 0.0001), the highest percentage of carcasses without bruises (*p* < 0.0001) and the lowest percentage of handling-type carcass bruises (*p* < 0.0001) (Table 10).

## 4. Discussion

This study assessed the influence of different transport conditions on antemortem and postmortem welfare indicators of slaughter pigs with different health status and RYR-1 genotype. There are some limitations of the present investigation. The key limitations of the present study are the low diagnostic specificity of the diagnostic protocol for subclinical pathological alterations (non-pathognomonic for a particular disease) and carcass bruises and the subjectivity of the visual assessment. Moreover, subclinical pathological lesions that develop early in the fattening period may undergo healing by the time of slaughter, making them difficult or even impossible to detect at the slaughterline. In addition, a significant limitation is the possibility that some of the examined subclinical pathological and carcass bruises occur as a result of artefacts, potentially arising from the processing of the carcass. Furthermore, the present study did not document the severity or extent of pathological lesions, a factor that could influence both ante- and postmortem welfare indicators. An important limitation of this investigation is the lack of information on other postmortem lesions (lung scarring, abscesses, tail-biting lesions, gastric ulcers etc.) that might also negatively affect examined variables.

### 4.1. Behavioural Recordings, Rigor Mortis and Carcass Bruises

The present investigation revealed that the group of slaughter pigs (Shipment 8), predominantly consisting of stress-carrier (Nn genotype, 56.67%) and subclinically diseased (60.00%) individuals, that were exposed to short transportation (<30 min) at high loading density (~235 kg/m^2^) had the highest percentage of slipping, falling, turning back and reluctance to move at unloading (Table 3 and Table 4). Previous studies reported that pigs carrying the mutant n allele show a rougher acute stress reaction to short transportation at high loading density and greater fatigue on arrival at the abattoir [11,17]. In addition, it is well known that Nn pigs exhibit greater anxiety and sensitivity to stressful stimuli than NN pigs, so they were more difficult to handle and needed more force during unloading, which resulted in rough handling [28,45]. As unloading facilities were the same for all audited shipments, it could be speculated that subclinically diseased pigs containing this detrimental allele exposed to extremely harsh pre-slaughter conditions were severely stressed and fatigued at unloading. Because those pigs were not easy to drive, abattoir personnel were nervous and frustrated, which led to rougher handling, more frequent use of electric prods and forcing them to move faster, thus provoking severe stress and more physical reactions such as slipping, falling and turning back during unloading. As supporting evidence of this notion, subclinically diseased Nn pigs exposed to short transportation (<30 min) (Table 10) and high loading density (~235 kg/m^2^) (Table 7) had the most developed rigor mortis (the smallest foreleg angle) and the highest percentage of severe carcass bruises and handling-type carcass bruises.

However, when a group of slaughter pigs (Batch 5) predominantly consisting of stress-carrier (Nn genotype, 56.67%) and subclinically diseased (73.33%) individuals was subjected to long transportation (>3.5 h) at low loading density (~203 kg/m^2^), significantly lower frequencies of slipping, falling, turning back and reluctance to move at unloading were recorded than in the previously mentioned case (Table 3 and Table 4). It could be assumed that when subclinically diseased Nn pigs have more time and sufficient floor space in the lorry to lie down during the transportation, they experience lower stress during the journey, which results in lower percentage of irregular behavioural reactions at unloading.

On the other hand, in the group consisting of stress-resistant (100%) and predominantly healthy (60%) pigs that underwent short transportation (<30 min) at high loading density (~235 kg/m^2^) (Shipment 3), none of the individuals showed irregular behavioural reactions during unloading (Table 3 and Table 4). It could be hypothesised that pigs of the NN genotype, even those that are subclinically diseased, are less sensitive to pre-slaughter treatment and are able to keep the same walking speed as the rest of the group. Therefore, those pigs were driven with less difficulty (showed by minor duration; Table 1) during unloading, which resulted in less interventions and gentle handling by abattoir personnel and, consequently, the absence of pigs’ fear, expressed by slipping, falling, turning back and reluctance to move. This is further confirmed by the present study, where healthy NN pigs exposed to short transportation (<30 min) (Table 10) and high loading density (~235 kg/m^2^) (Table 7) had the least developed rigor mortis (the biggest foreleg angle), the highest percentage of carcasses without bruises and the lowest percentage of handling-type carcass bruises.

In the present study, the highest percentages of panting and shivering at unloading were observed after short transportation (<30 min) at high loading density (~235 kg/m^2^) in a group of slaughter pigs predominantly consisting of stress-carrier (Nn genotype, 56.67%) and subclinically diseased (60.00%) individuals (Shipment 8) (Table 3 and Table 4). Considering that ambient temperatures (14.5–21.0 °C) and temperature-humidity indices (59.22–66.13) during the investigation were within the thermoneutral zone (10–21 °C; [9]) and below the recommended threshold for heat alert (70–74; [46]) for slaughter pigs, expression of thermal behaviours at unloading could be ascribed to the facts that 60.00% and 56.67% of slaughter pigs from Shipment 8 had lung lesions and mutant n allele, respectively (Table 3). Exposing stress-carrier pigs to inappropriate transport conditions, such as transportation over short distances at high loading density, can trigger the onset of porcine stress syndrome, manifested by laboured and irregular breathing, hyperthermia, muscle and tail tremors, skin blanching and reddening, collapse, muscle rigidity and eventual death [47]. In addition, panting is a behavioural sign of poor physical fitness, which, in combination with increased occurrence of lung lesions in these pigs, leads to lower respiration rate and difficulty in meeting oxygen demand after a physical effort [48]. Hence, the synergistic effect of inappropriate transport conditions, subclinical respiratory disease and higher sensitivity to stress in slaughter pigs containing the mutant n allele resulted in the expression of thermal behaviours at unloading, such as panting (laboured breathing) and shivering (muscle tremors).

### 4.2. Serum Metabolites

Analysis of stress metabolites revealed that subclinically diseased Nn pigs subjected to short transportation (<30 min) (Table 9) and high loading density (~235 kg/m^2^) (Table 6) had the highest blood concentration of lactate and glucose, indicating their higher susceptibility, greater fatigue and seriously compromised welfare on the day of slaughter. A higher predisposition for accumulation of lactate and glucose in the bloodstream of stress-carrier individuals (Nn genotype) can be explained by the fact that the genetic selection of pigs for improved lean meat content has increased the number of glycogen-rich white muscle fibres, resulting in two- to three-times greater muscle glycogen stores than in stress-resistant pigs (NN genotype) [49,50,51]. Also, a more rapid peri- and postmortem metabolism is expected in slaughter pigs with the RYR-1 mutation due to a higher proportion of fast twitch glycolytic fibres, lower proportion of the slow twitch oxidative type, increased fibre diameter and, thus, greater glycolytic metabolic potential in their skeletal muscles [17]. Therefore, after exposure of Nn pigs to short-term acute stress, such as short transportation and high loading density, the sympathetic–adrenal–medullary axis is activated, resulting in the secretion of catecholamines, including noradrenaline and adrenaline. Adrenergic stress response leads to accelerated glycogenolysis in skeletal muscle and liver, and consequently, elevated blood lactate and glucose concentrations [52,53]. In addition, a diminished lung capacity resulting from pathological alterations contributes to reduced oxygen transfer to arterial blood. Simultaneously, an elevated respiratory rate increases oxygen consumption, prompting a quicker shift to anaerobic metabolism in skeletal muscles [54]. This results in disbalance between anaerobic and aerobic metabolism and leads to heightened lactate production coupled with a diminished ability to eliminate it [54,55]. This can further explain the highest blood glucose and lactate levels found in subclinically diseased Nn pigs that underwent short transportation (<30 min) (Table 9) at high loading density (~235 kg/m^2^) (Table 6), since all individuals (100%) had lung lesions (Table 5 and Table 8). Accordingly, it can be argued that an additive effect between genotype and health status aggravated the negative influence of short transportation at high loading density, with RYR-1 gene carrier and subclinically diseased pigs showing a greater fatigue and rougher stress reaction to these transport conditions.

In cases when subclinically diseased Nn pigs were exposed to long transportation (>3.5 h) (Table 9) and low loading density (~203 kg/m^2^) (Table 6), significantly lower blood lactate and glucose levels were recorded, indicating milder stress reaction and better welfare conditions. It has been reported that when sufficient lorry floor space is available, most slaughter pigs, regardless of RYR-1 genotype and health status, begin to sit and lie down during the first 30 min of the journey [7,56]. Accordingly, when provided more transportation time (>3.5 h) with sufficient floor space (~0.50 m^2^/100 kg), even subclinically diseased stress-carrier pigs have enough time and space to lie down, calm down, rest, partially recover from the stress induced during loading and transportation and, to a certain extent, acclimate to transport conditions, which, all together, positively affect pig welfare.

On the other hand, healthy NN pigs exposed to short transportation (<30 min) (Table 9) and high loading density (~235 kg/m^2^) (Table 6) had the lowest blood levels of lactate and glucose, which were within or close to the reference range for slaughter pigs. This indicates that healthy stress-resistant pigs had the ability to cope with short-term acute stress, especially when provided the optimal floor space in the lorry suggested by EU regulations (235 kg/m^2^; [30]), which allow them to stand or lie down. Therefore, it can be argued that those pigs were not fatigued and physically stressed after being exposed to short transportation (<30 min) and high loading density (~235 kg/m^2^), and thus, their welfare was not compromised.

### 4.3. Serum Hormones

Despite the fact that, regardless of transport conditions, genotype and health status, cortisol levels in all groups of slaughter pigs were much higher than the basal levels of the species, no significant effects of pre-slaughter conditions were found (*p* > 0.05; Table 6 and Table 9) on plasma ACTH and cortisol concentrations. Comparable results were reported by Weaver et al. [57], Yoshioka et al. [58] and Averos et al. [11], who demonstrated that the presence of the RYR-1 deleterious allele did not affect concentration of the aforementioned stress hormones. Also, it has been reported that presence of subclinical pathological lesions had no influence on the concentration of ACTH and cortisol [59]. Although several studies have demonstrated that pig transportation could have induced hypercortisolemia [60] and elevated levels of ACTH [61], the results are not consistent. This can be explained by the fact that that several factors of variation (e.g., circadian rhythm, breed, genotype, gender, age, feeding regimen and repeatability for the same stressors) may affect levels of stress hormones [61]. Based on the results obtained in this study, it can be argued that cortisol and ACTH have limited uses as physiological biomarkers of stress intensity experienced by slaughter pigs with different RYR-1 genotype and health status during various transport conditions.

### 4.4. Electrolytes

Analysis of plasma electrolytes revealed that slaughter pigs, regardless of transport conditions, RYR-1 genotype and health status, had plasma sodium and chloride levels below the reference range for the species (Table 6 and Table 9), indicating some degree of dehydration in most of individuals during the pre-slaughter period. In addition, the lowest plasma concentrations of sodium and chloride were recorded in subclinically diseased Nn pigs exposed to short transportation (<30 min) (Table 9) and high loading density (~235 kg/m^2^) (Table 6), suggesting the highest dehydration level. Dehydration in slaughter pigs can be a result of pathological fluid loss, diminished feed and water intake, stress or all of those reasons occurring simultaneously [62,63]. Taking into account all pre-slaughter conditions, the high dehydration level in the aforementioned group could be ascribed to the synergistic effect of stressful transport treatment, prolonged food and water deprivation, increased susceptibility to stress (deleterious n allele) and poor health status. After exposing Nn pigs with lung lesions to transport stress, body temperature and respiratory rate markedly increase [58], which causes rapid, open-mouth panting, enhancing evaporative heat loss, but leads to the occurrence of negative consequences, such as dehydration and respiratory alkalosis [64]. As previously mentioned, the highest frequency of panting was recorded in a group of slaughter pigs, predominantly consisting of stress-carrier (Nn genotype, 56.67%) and subclinically diseased (60.00%) individuals (Shipment 8), who were subjected to short transportation (<30 min) at high loading density (~235 kg/m^2^) (Table 3 and Table 4), which can further explain the poor hydration state. Although some degree of dehydration is an unavoidable consequence on the day of slaughter due to withholding of water and feed [53], this can be further aggravated by poor health status together with increased stress susceptibility. Hence, if unhealthy Nn pigs are included in the transport, good hydration is of paramount importance, and it is recommended to provide sufficient amounts of more readily available water to maximise animal welfare [52,53].

In cases when subclinically diseased Nn pigs underwent long transportation (>3.5 h) (Table 9) and low loading density (~203 kg/m^2^) (Table 6), significantly lower plasma sodium and chloride levels were recorded than in the aforementioned group, suggesting a better dehydration state. It can be assumed that when the available floor area in the lorry is increased by 20%, subclinically diseased Nn pigs have no need to fight with the surrounding pigs for space, and therefore, they are less stressed, less fatigued and lose less water through panting, physical activities and confrontations during longer transportation, which, all together, positively affects their hydration state.

In contrast, healthy NN pigs exposed to short transportation (<30 min) (Table 9) and high loading density (~235 kg/m^2^) (Table 6) had the highest plasma sodium and chloride concentrations, which were within or close to the ranges considered normal for slaughter pigs, showing that those individuals were in a good hydration state. As mentioned earlier, in the group consisting of stress-resistant (100%) and predominantly healthy (60%) pigs that underwent short transportation (<30 min) at high loading density (~235 kg/m^2^) (Shipment 3), none of the individuals showed panting during unloading (Table 3 and Table 4). This implies that those pigs do not lose body water due to enhanced evaporation through panting, which can further explain the good hydration state of the predominantly healthy stress-resistant slaughter pigs transported over short distances at a loading density permissible under EU legislation (0.425 m^2^/100 kg).

### 4.5. Stress Enzymes

The results of the stress enzyme analysis obtained in this study revealed that subclinically diseased Nn pigs exposed to short transportation (<30 min) (Table 9) and high loading density (~235 kg/m^2^) (Table 6) had the highest plasma activities of CK, LDH, AST and ALT. The obtained results can be ascribed by the fact that the large majority of individuals from this groups of pigs had lung lesions (100.00), liver milk spots (92.00%), pericarditis (36.00%) and severe (84.00%) carcass bruises (Table 5, Table 7, Table 8 and Table 10). The increase in stress enzymes activity in slaughter pigs occur when skeletal muscles, lung, liver, pancreas and/or heart are damaged [15,43,53,59,65,66], and therefore, several possible explanations can be provided for the obtained results. In cases of extremely high levels of muscle activity and muscular damage as a result of a higher proportion of injuries on the day of slaughter, CK, LDH, AST and ALT are released into the circulation due to disruptions in the muscle cell membrane and cell permeability [66]. Additionally, increased CK activity in pigs can be linked with strenuous respiratory muscle activity and higher breathing rate during respiratory disorders [67] and in the case of heart injury during transportation or the presence of heart disease (pericarditis) [65,68]. The elevation in LDH activity in the bloodstream is indicative of lung epithelium damage, as lung tissue is abundant in LDH. This increase suggests the release of LDH from epithelial cells that line the airways [69]. Furthermore, increased concentrations of AST and ALT may be an indication of hepatic damage (in the form of milk spots in the liver), caused by the migrating *Ascaris suum* larvae and consequent leakage of these enzymes into the extracellular space and, subsequently, into the circulation [70]. Another possible explanation is that stress-carrier pigs (Nn genotype) have higher lean meat content [28] and, thus, greater potential for an increase in stress enzymes’ activity in the circulation due to fact that skeletal muscles are the main sources of CK, LDH, AST and ALT [50].

When subclinically diseased Nn pigs were exposed to long transportation (<3.5 h) (Table 9) and low loading density (~200 kg/m^2^) (Table 6), significantly lower plasma activities of stress enzymes were recorded than in the previously mentioned case. It can be hypothesised that the lower loading density during a longer journey may allow subclinically diseased Nn pigs the opportunity to escape from sources of aggression of other conspecifics, and, after a settling time, provide sufficient floor space and time for all pigs to lie and rest. This is confirmed by the results of this study, where subclinically diseased Nn pigs subjected to long transportation (<3.5 h) and low loading density (~200 kg/m^2^) had 1.5- and 2.5-times lower frequencies of severe and fighting-type carcass bruises than those exposed to short transportation (<30 min) and high loading density (~235 kg/m^2^) (Table 7 and Table 10). This can explain the lower activities of plasma CK, LDH, AST and ALT found in subclinically diseased Nn pigs subjected to long transportation (<3.5 h) and low loading density (~200 kg/m^2^).

On the other hand, healthy NN pigs exposed to short transportation (<30 min) (Table 9) and high loading density (~235 kg/m^2^) (Table 6) had the lowest plasma CK and LDH activities. Based on the results obtained in this study, it can be argued that healthy stress-resistant slaughter pigs exposed to short distance transportation (~5 km) at a loading density permissible under EU legislation (0.425 m^2^/100 kg) experienced only mild physical stress and tissue damage. Additionally, in healthy NN pigs exposed to short transportation (<30 min) (Table 9) and high loading density (~235 kg/m^2^) (Table 6), plasma AST and ALT levels were in the ranges considered normal for slaughter pigs, while plasma levels of CK and LDH were close (slightly above) to the basal levels of the species, confirming the lack of any serious pathological condition and injuries in those individuals.

### 4.6. Acute Phase Proteins

Earlier investigations have reported that transportation [12,71,72,73] and occurrence of sub(clinical) diseases in pigs [59,74,75,76] could cause a significant acute-phase response. In this study, the highest plasma ceruloplasmin concentrations but the lowest plasma albumin and PON-1 levels were recorded in subclinically diseased Nn pigs exposed to short transportation (<30 min) (Table 9) and high loading density (~235 kg/m^2^) (Table 6), indicating the strongest activation of acute phase response. In addition, in these groups, plasma albumin levels were slightly below the reference values for slaughter pigs, while CRP and haptoglobin levels were far below the basal levels of the species (Table 6 and Table 9). Unexpectedly low plasma haptoglobin and CRP levels in combination with high plasma ceruloplasmin levels in the aforementioned group of pigs can be explained by the fact that during acute phase response, synthesis of ceruloplasmin is more delayed than that of CRP or haptoglobin [77], indicating the longer-lasting disease and prolonged stress reaction. This is further confirmed by the fact that 48.00% and 28.00% of slaughter pigs from this group had subclinical pathological lesions in two and three organ systems, respectively (Table 5 and Table 8), confirming the longer-lasting disease in these individuals. In addition to the liver, almost the same amount of ceruloplasmin is synthesised in the lungs [78], where its serum concentrations rapidly increase during respiratory infections and heart diseases [79,80]. Therefore, another explanation for high plasma ceruloplasmin concentration is the fact that the large majority of subclinically diseased Nn pigs exposed to short transportation (<30 min) and high loading density (~235 kg/m^2^) had lung lesions (80.00%), liver milk spots (52.00%) and pericarditis (12.00%) (Table 5 and Table 8).

However, when subclinically diseased Nn pigs underwent long transportation (>3.5 h) (Table 9) at low loading density (~203 kg/m^2^) (Table 6), lower plasma ceruloplasmin concentrations but higher plasma albumin and PON-1 levels were recorded compared to the aforementioned case, indicating significantly lower acute phase response. As previously emphasised, during longer journeys (>3 h) with more available floor space in the lorry (~0.50 m^2^/100 kg), even subclinically diseased stress-carrier pigs experience milder stress, and therefore, lower concentrations of acute phase proteins are expected.

Under normal circumstances, i.e., when pigs are not sick and/or under stress, they are able to maintain normal body homeostasis and metabolism. Therefore, in such cases, undetectable or low levels of positive acute phase proteins and physiological or high levels of negative acute phase proteins can be expected, which are indicators of good health and welfare status in slaughter pigs [43,59,81,82]. The present investigation revealed that healthy NN pigs exposed to short transportation (<30 min) (Table 9) and high loading density (~235 kg/m^2^) (Table 6) had the lowest plasma ceruloplasmin concentration and the highest levels of albumin and PON-1. The aforementioned group of pigs had extremely low plasma CRP and haptoglobin concentrations and plasma albumin levels within the reference range for slaughter pigs (Table 6 and Table 9), indicating absence of the acute phase response. Accordingly, the obtained results further confirmed that those pigs were not under intensive stress and lacked any serious pathological condition, suggesting good health and welfare status.

### 4.7. Oxidative Stress Biomarkers

In the present study, the highest MDA production was recorded in subclinically diseased Nn pigs exposed to short transportation (<30 min) (Table 9) and high loading density (~235 kg/m^2^) (Table 6), indicating impaired antioxidant defence and induction of oxidative stress. In addition, the same group of pigs had the lowest GSH production (Table 6 and Table 9), which indicates that these antioxidants were over-consumed in the detoxification of free radicals and further confirms the activation of oxidative stress. Any kind of stress, including transportation and/or disease, causes an imbalance between the antioxidant and oxidant levels in the organism and creates an environment in which cells undergo oxidative stress [16]. Therefore, it can be speculated that the activation of oxidative stress resulted from the simultaneous effects of stressful transport conditions, subclinical diseases and higher sensitivity to stress. Some authors [17] demonstrated less potent antioxidant defences in pigs carrying the detrimental n allele, while others [16] reported a significant increase in MDA production after short transportation in pigs. In addition, the occurrence of oxidative stress has been also implicated in numerous disease processes in pigs, and oxidative parameters have been proposed as biomarkers to identify animals at risk of diseases [83].

On the other hand, when subclinically diseased Nn pigs underwent long transportation (>3.5 h) (Table 9) and low loading density (~203 kg/m^2^) (Table 6), lower plasma MDA concentrations but higher plasma GSH levels were recorded compared to the previous group, implying reduced oxidative stress response. This further confirmed all other results obtained in this study, namely that more available space and time in the lorry for subclinically diseased stress-carrier pigs resulted in significantly lower stress response and, in turn, improved pig welfare.

In contrast, healthy NN pigs exposed to short transportation (<30 min) (Table 9) and high loading density (~235 kg/m^2^) (Table 6) had the lowest MDA production and the highest GSH production, suggesting higher antioxidant defence and absence of oxidative stress. Therefore, up-regulation of GSH in the aforementioned group of pigs might be an adaptation mechanism during stressful situations to prevent cellular damage or counteract oxidative stress.

## 5. Conclusions

The results of this study showed the different degree of alterations in behavioural indicators and physiometabolic blood profile depending on transport conditions, genotype and health status of slaughter pigs. The most compromised welfare was recorded in subclinically diseased pigs carrying a mutant n allele exposed to short transportation (<30 min) and high loading density (~235 kg/m^2^), shown through irregular behavioural reactions at unloading, the greatest fatigue, the highest dehydration level, severe physical stress and tissue damage, the strongest acute phase response and initiation of oxidative stress. Compared to the previous group, subclinically diseased Nn pigs subjected to long transportation (<3.5 h) and low loading density (~200 kg/m^2^) showed lower frequencies of irregular behavioural reactions at unloading, lower fatigue, better hydration state, lower physical stress and lower acute phase and oxidative stress response. This indicates that subclinically diseased stress-carrier pigs require more time in transit and about 20% more floor space in the lorry for improvement of their well-being. On the contrary, the welfare of healthy NN pigs was not compromised following short transportation (<30 min) and high loading density (~235 kg/m^2^), shown through normal behavioural reactions during unloading, only mild fatigue, negligible physical stress and tissue damage, good hydration state and the absence of acute phase response and oxidative stress. Based on the obtained results, it can be concluded that stress-carrier pigs with subclinical pathological lesions should not be considered fit for transportation, indicating that the health status and genotype are the key factors for optimising pig welfare over short transport distances at the optimal loading density (0.425 m^2^/100 kg). An assessment of fitness for journey should be performed to avoid transporting slaughter pigs with subclinical/clinical diseases. Therefore, valid methods that can be used to identify the slaughter pigs that are most susceptible to disease or subclinically diseased while in the farm of origin are needed. Guaranteeing the welfare of slaughter pigs throughout the phases preceding slaughter requires good health status (reduction of lung lesions by adequate vaccination and mandatory parasite control in optimal time intervals during raising), appropriate genetics (elimination of mutant n allele from pig populations), optimal ambient conditions and access to water and food, while respecting appropriate transportation time and loading density with comfortable lairage conditions. Additional research is necessary to determine the effects of different transportation factors, such as weather conditions, loading density and transportation time and their interaction, on behaviour, physiology and carcass and meat quality in slaughter pigs with different health status and RYR-1 genotype.

## Figures and Tables

**Table 1:** The data regarding pre-slaughter conditions of the eight shipments of slaughter pigs included in the study (*n =* 240).

Shipment	1	2	3	4	5	6	7	8
General information								
Number of pigs	30	30	30	30	30	30	30	30
Farm of origin	A	B	A	B	A	B	A	B
Season	Spring	Spring	Spring	Spring	Spring	Spring	Spring	Spring
Month	April	April	April	April	May	May	May	May
Loading conditions								
Loading time (minutes)	63	45	35	60	58	63	70	82
Loading density (kg/m^2^)	200	198	236	236	203	200	235	235
Ambient temperature at loading (°C)	15.4	14.5	16.5	18.0	21.0	19.5	20.6	18.9
Ambient relative humidity at loading (%)	42.0	57.3	60.0	42.8	63.0	57.1	48.4	53.3
Temperature-humidity index at loading	59.32	58.56	60.48	62.89	65.72	64.24	66.13	63.67
Transport conditions								
Transportation time (minutes)	225	22	245	21	210	25	230	23
Transport distance (km)	215	5	215	5	215	5	215	5
Unloading conditions								
Waiting time (minutes)	10	25	5	20	16	8	14	7
Unloading time (minutes)	33	20	10	35	29	30	44	65
Ambient temperature at unloading (°C)	16.5	15.5	17.7	18.7	19.5	18.0	21.0	20.5
Ambient relative humidity at unloading (%)	56.70	65.50	54.50	62.80	58.40	50.00	60.00	47.30
Temperature-humidity index at unloading	60.55	59.22	62.10	63.01	63.20	62.64	65.91	66.06
Lairage conditions								
Lairage time (minutes)	67	60	62	65	68	60	63	64
Lairage density (m^2^/pig)	0.65	0.65	0.65	0.65	0.65	0.65	0.65	0.65
Lairage temperature (°C)	18.50	16.6	17.6	19.3	20.5	19.0	20.0	19.20
Lairage relative humidity (%)	60.00	55.80	59.30	62.2	50.00	52.70	63.50	48.80
Temperature-humidity index at lairage	62.89	60.69	61.83	61.30	65.78	63.28	64.51	64.26

**Table 2:** Antemortem welfare indicators recorded during unloading and lairage at the abattoir.

Checkpoint	Behaviour	Description
U	Slipping	Loss of balance without the body touching the floor.
U	Falling	Loss of balance in which a part of the body other than the legs are in contact with the floor.
U	Turning back	Pig facing towards the unloading zone makes a 180° turn in the direction of the lorry area.
U	Reluctance to move	Pig showing reluctance to move, having stopped walking, without moving its head and body, and failed to explore for at least 2 s.
U	Lameness	Inability to use one or more limbs in a normal manner.
U, L	Panting	Short gasps carried out with the mouth when the pig is breathing rapidly.
U, L	Shivering	Slow and irregular vibration of any body part, or the body as a whole.
L	Huddling	Pig lying with more than half of its body in contact with another pig.
L	Coughing	Pigs displaying an audible expulsion of air through the mouth.
L	Sneezing	Pigs displaying sudden involuntary expulsion of air from the nose and mouth due to irritation of the nostrils.
U	Sick animals	Pigs that are unable to walk and/or keep up with the rest of the group.
U, L	Dead animals	-

**Table 3:** Differences in frequency of RYR-1 genotypes and health status of slaughter pigs in relation to transport conditions at the shipment level (*n =* 240).

	Shipment 1	Shipment 2	Shipment 3	Shipment 4	Shipment 5	Shipment 6	Shipment 7	Shipment 8	Chi-Square	df	*p*-Value
Number of pigs	30	30	30	30	30	30	30	30			
Transport conditions											
Loading density	Low	Low	High	High	Low	Low	High	High	-	-	-
Transportation time	Long	Short	Long	Short	Long	Short	Long	Short	-	-	-
RYR-1 genotype											
NN pigs (%)	56.67	90.00	100.00	60.00	43.33	53.33	63.33	43.33	-	-	-
Nn pigs (%)	43.33	10.00	0.00	40.00	56.67	46.67	36.67	56.67	-	-	-
Health status											
Healthy pigs (%)	46.67	43.33	60.00 ^a^	20.00 ^b^	26.67 ^b^	23.33 ^b^	46.67	40.00	16.92	7	0.0179
Subclinically diseased pigs (%)	53.33	56.67	40.00 ^a^	80.00 ^b^	73.33 ^b^	76.67 ^b^	53.33	60.00	16.92	7	0.0179
Lung lesions (%)	50.00	43.33	40.00	70.00	60.00	70.00	50.00	60.00	11.05	7	0.1366
Liver milk spots (%)	20.00 ^a^	36.67 ^a^	6.67 ^b^	60.00 ^a^	56.67 ^a^	33.33 ^a^	43.33 ^a^	50.00 ^a^	32.58	7	<0.0001
Pericarditis (%)	0.00	3.33	3.33	13.33	6.67	16.67	16.67	16.67	11.88	7	0.1047
Lesions in one organ system (%)	36.66	30.00	30.00	43.33	30.00	46.67	16.66	16.66	11.77	7	0.1083
Lesions in two organ systems (%)	16.67	26.67	10.00	33.33	36.66	20.00	20.00	26.67	9.00	7	0.2529
Lesions in three organ systems (%)	0.00	0.00	0.00	3.34	6.67	10.00	16.67	16.67	17.14	7	0.0165

Abbreviations: high loading density—space allowance in the lorry within or slightly lower than recommended density of ~235 kg/m^2^; low loading density—the space allowance in the lorry higher than ~235 kg/m^2^; short transportation—slaughter pigs transported for less than 30 min; long transportation—slaughter pigs transported for more than 3.5 h; NN pigs—stress-resistant; Nn pigs—stress-carrier; healthy pigs—slaughtered pigs without clinical signs of disease at antemortem examination and pathological lesions in lungs, liver and heart at postmortem examination; subclinically diseased pigs—slaughtered pigs without clinical signs of disease at antemortem examination but with any sign of pathological lesions in lungs, liver and heart at postmortem examination; lung lesions—slaughtered pigs with any sign of pneumonia and pleurisy; liver milk spots—slaughtered pigs with at least one milk spot lesion in liver; pericarditis—slaughtered pigs with adhesion between the heart and the pericardium; df—degrees of freedom. Note: different letters in the same row indicate a significant difference at *p* < 0.05 ^(a,b)^.

**Table 4:** Differences in antemortem welfare indicators, frequency of RYR-1 genotypes and health status in relation to eight shipments of slaughter pigs included in the study (*n =* 240).

Antemortem Welfare Indicators	Shipment 1	Shipment 2	Shipment 3	Shipment 4	Shipment 5	Shipment 6	Shipment 7	Shipment 8	Chi-Square	df	*p*-Value
Number of pigs	30	30	30	30	30	30	30	30			
Unloading area											
Slipping (%)	26.67 ^ac^	20.00 ^bc^	0.00 ^b^	30.00 ^ac^	20.00 ^a^	20.00 ^a^	43.33 ^c^	80.00 ^d^	60.19	7	<0.0001
Falling (%)	10.00 ^a^	10.00 ^a^	0.00 ^a^	10.00 ^a^	6.67 ^a^	6.67 ^a^	10.00 ^a^	36.67 ^b^	24.66	7	0.0009
Turning back (%)	3.33 ^a^	0.00 ^a^	0.00 ^a^	3.33 ^a^	0.00 ^a^	6.67 ^a^	6.67 ^a^	30.00 ^b^	35.77	7	<0.0001
Reluctance to move (%)	0.00 ^a^	0.00 ^a^	0.00 ^a^	0.00 ^a^	0.00 ^a^	3.33 ^a^	3.33 ^a^	23.33 ^b^	37.75	7	<0.0001
Lameness (%)	0.00	0.00	0.00	0.00	0.00	0.00	3.33	6.67	10.46	7	0.1638
Panting (%)	0.00 ^a^	0.00 ^a^	0.00 ^a^	0.00 ^a^	0.00 ^a^	0.00 ^a^	16.67 ^b^	33.33 ^c^	57.17	7	<0.0001
Shivering (%)	0.00 ^a^	0.00 ^a^	0.00 ^a^	0.00 ^a^	0.00 ^a^	0.00 ^a^	3.33 ^a^	26.67 ^b^	50.68	7	<0.0001

Abbreviations: df—degrees of freedom. Note: different letters in the same row indicate a significant difference at *p* < 0.05 ^(a–c)^.

**Table 5:** Differences in pathological lesions in slaughter pigs in relation of loading density, RYR-1 genotype and health status (*n =* 240).

Loading Density	High				Low						
RYR-1 Genotype	NN		Nn		NN		Nn				
Health Status	Healthy	Diseased	Healthy	Diseased	Healthy	Diseased	Healthy	Diseased	Chi-Square	df	*p*-Value
Number of pigs	35	45	15	25	24	49	18	29			
Type of pathological lesions											
Lung lesions (%)	0.00 ^a^	88.89 ^b^	0.00 ^a^	100.00 ^bc^	0.00 ^a^	77.55 ^ab^	0.00 ^a^	96.55 ^bc^	183.8	7	<0.0001
Liver milk spots (%)	0.00 ^a^	53.33 ^b^	0.00 ^a^	92.00 ^c^	0.00 ^a^	59.18 ^b^	0.00 ^a^	51.72 ^b^	103.6	7	<0.0001
Pericarditis (%)	0.00 ^a^	13.33 ^b^	0.00 ^a^	36.00 ^c^	0.00 ^a^	10.20 ^b^	0.00 ^a^	10.34 ^b^	30.66	7	<0.0001
Spread of pathological lesions											
Lesions in one organ system (%)	0.00 ^a^	57.78 ^b^	0.00 ^a^	24.00 ^c^	0.00 ^a^	59.18 ^b^	0.00 ^a^	44.83 ^b^	78.01	7	<0.0001
Lesions in two organ systems (%)	0.00 ^a^	33.33 ^b^	0.00 ^a^	48.00 ^b^	0.00 ^a^	34.69 ^b^	0.00 ^a^	48.27 ^b^	51.29	7	<0.0001
Lesions in three organ systems (%)	0.00 ^a^	8.89 ^a^	0.00 ^a^	28.00 ^b^	0.00 ^a^	6.13 ^a^	0.00 ^a^	6.90 ^a^	25.24	7	0.0007

Abbreviations: high loading density—space allowance in the lorry within or slightly lower than recommended density of 235 kg/m^2^; low loading density—the space allowance in the lorry higher than 235 kg/m^2^; NN pigs—stress-resistant; Nn pigs—stress-carrier; healthy pigs—slaughtered pigs without clinical signs of disease at antemortem examination and pathological lesions in lungs, liver and heart at postmortem examination; diseased pigs—slaughtered pigs without clinical signs of disease at antemortem examination but with any sign of pathological lesions in lungs, liver and heart at postmortem examination; Lung lesions—slaughtered pigs with any sign of pneumonia and pleurisy; liver milk spots—slaughtered pigs with at least one milk spot lesion in liver; pericarditis—slaughtered pigs with adhesion between the heart and the pericardium; df—degrees of freedom. Note: different letters in the same row indicate a significant difference at *p* < 0.05 ^(a–c)^.

**Table 6:** Influence of loading density on the physiometabolic blood profile of slaughter pigs with different health status and RYR-1 genotype (*n =* 240).

Loading Density (LD)	High				Low									
RYR-1 Genotype (G)	NN		Nn		NN		Nn				Main Effects			Interaction
Health Status (HS)	Healthy	Diseased	Healthy	Diseased	Healthy	Diseased	Healthy	Diseased	SEM	Reference Values	LD	G	HS	LD × G × HS
Number of pigs	35	45	15	25	24	49	18	29		[43]	*p*-Value			
Stress metabolites														
Lactate (mmol/L)	5.83 ^a^	10.35 ^b^	21.14 ^c^	23.30 ^d^	14.69 ^e^	17.64 ^f^	12.92 ^g^	17.16 ^f^	0.79	0.50–5.50	0.0245	<0.0001	<0.0001	<0.0001
Glucose (mmol/L)	5.03 ^a^	7.94 ^b^	9.72 ^c^	14.90 ^d^	7.80 ^b^	8.52 ^bc^	7.33 ^b^	8.49 ^bc^	0.91	3.70–6.40	<0.0001	<0.0001	<0.0001	0.0450
Stress hormones														
Cortisol (nmol/L)	244.32	250.85	243.83	256.00	289.84	301.96	285.68	277.43	68.20	76.00–88.00	0.0867	0.7912	0.8037	0.775
ACTH (pmol/L)	0.87	0.80	1.47	1.55	0.56	0.68	0.33	0.27	0.87	-	0.0522	0.5558	0.9556	0.7860
Electrolytes														
Sodium (mmol/L)	140.58 ^a^	130.07 ^b^	123.00 ^c^	117.33 ^d^	129.53 ^b^	126.32 ^bc^	130.0 ^bc^	127.07 ^bc^	2.17	140.00–150.00	0.5771	<0.0001	<0.0001	<0.0001
Chloride (mmol/L)	91.09 ^a^	84.35 ^b^	82.86 ^b^	79.47 ^c^	85.50 ^b^	85.70 ^b^	85.63 ^b^	84.36 ^b^	1.83	94.00–103.00	0.1916	<0.0001	<0.0001	0.0001
Stress enzymes														
CK (units/L)	1461.93 ^a^	2353.00 ^b^	3509.50 ^c^	4263.12 ^d^	3406.60 ^c^	3499.43 ^c^	2932.57 ^bc^	3721.25 ^c^	303.47	66.00–489.00	<0.0001	<0.0001	<0.0001	<0.0001
LDH (units/L)	873.59 ^a^	1452.31 ^b^	3224.44 ^c^	4497.21 ^d^	1710.41 ^e^	1750.32 ^e^	2269.00 ^f^	2139.09 ^f^	356.33	380.00–630.00	<0.0001	<0.0001	0.0005	<0.0001
AST (units/L)	46.00 ^a^	41.00 ^b^	41.50 ^b^	86.73 ^c^	30.77 ^d^	34.58 ^e^	36.62 ^e^	34.36 ^e^	15.40	32.00–84.00	0.0013	0.0534	0.0844	0.0208
ALT (units/L)	33.65 ^a^	33.84 ^a^	42.80 ^b^	58.13 ^c^	34.60 ^a^	35.27 ^a^	34.13 ^a^	37.53 ^a^	4.14	31.00–58.00	<0.0001	<0.0001	0.0024	0.0500
Acute-phase proteins														
Haptoglobin (mg/L)	0.29	0.25	0.32	0.29	0.20	0.26	0.20	0.23	0.08	20.00–3000.00	0.0511	0.6068	0.9095	0.7720
CRP (mg/L)	1.06	1.00	1.00	1.17	1.00	1.00	1.00	1.12	0.14	5.00–30.00	0.5814	0.2628	0.2628	0.9810
Albumin (g/L)	40.19 ^a^	33.92 ^b^	30.93 ^c^	25.96 ^d^	35.22 ^b^	33.94 ^b^	32.50 ^bc^	35.67 ^b^	1.28	30.00–40.00	0.0005	<0.0001	<0.0001	<0.0001
Ceruloplasmin (mg/dL)	10.79 ^a^	21.92 ^b^	36.44 ^c^	45.24 ^d^	25.53 ^e^	25.16 ^e^	21.13 ^b^	25.43 ^e^	3.24	-	<0.0001	<0.0001	<0.0001	0.0334
PON-1 (units/L)	1838.68 ^a^	967.07 ^b^	707.38 ^c^	236.79 ^d^	970.90 ^b^	841.24 ^e^	1045.03 ^b^	939.85 ^b^	173.27	-	<0.0001	<0.0001	<0.0001	0.0122
Oxidative stress biomarkers														
MDA (nmol/mL)	2.54 ^a^	3.64 ^b^	5.39 ^c^	8.73 ^d^	5.56 ^c^	5.22 ^c^	5.25 ^c^	5.77 ^c^	0.48	-	0.0019	<0.0001	<0.0001	0.0048
GSH (µM/L)	2.77 ^a^	1.48 ^b^	0.91 ^c^	0.46 ^d^	1.24 ^b^	1.07 ^c^	1.02 ^c^	1.02 ^c^	0.34	-	0.0002	<0.0001	<0.0001	0.0042

Abbreviations: high loading density—space allowance in the lorry within or slightly lower than recommended density of 235 kg/m^2^; low loading density—the space allowance in the lorry higher than 235 kg/m^2^; NN pigs—stress-resistant; Nn pigs—stress-carrier; healthy pigs—slaughtered pigs without clinical signs of disease at antemortem examination and pathological lesions in lungs, liver and heart at postmortem examination; diseased pigs—slaughtered pigs without clinical signs of disease at antemortem examination but with any sign of pathological lesions in lungs, liver and heart at postmortem examination; ACTH—adrenocorticotropic hormone; CK—creatine kinase; LDH—lactic dehydrogenase; AST—aspartate amino transferase; ALT—alanine amino transferase; CRP—C-reactive protein; MDA—Malondialdehyde; GSH—Glutathione; PON-1—paraoxonase-1; SEM—pooled standard error of means. Note: different letters in the same row indicate a significant difference at *p* < 0.05 ^(a–g)^.

**Table 7:** Influence of loading density on the rigor mortis and carcass bruises of slaughter pigs with different health status and RYR-1 genotype (*n =* 240).

Loading Density	High				Low					
RYR-1 Genotype	NN		Nn		NN		Nn			
Health Status	Healthy	Diseased	Healthy	Diseased	Healthy	Diseased	Healthy	Diseased	SEM	*p*-Value
Number of pigs	35	45	15	25	24	49	18	29		
Rigor mortis										
RM (°)	12.91 ^a^	117.24 ^b^	117.27 ^b^	110.40 ^c^	117.46 ^b^	116.51 ^b^	115.22 ^b^	116.59 ^b^	1.43	0.0018
Rigor score	1.66	1.91	1.60	1.72	1.88	1.55	1.39	1.62	0.45	0.130
Carcass bruise severity									Chi-square, df	
No carcass bruises	71.43 ^a^	20.00 ^bc^	13.33 ^bc^	8.00 ^b^	37.50 ^bc^	34.69 ^bc^	27.78 ^bc^	31.03 ^bc^	37.33, 7	<0.0001
Moderate carcass bruises	20.00 ^ab^	31.11 ^a^	40.00 ^a^	8.00 ^b^	25.00 ^ab^	34.69 ^a^	55.55 ^a^	17.24 ^ab^	17.03, 7	0.0172
Severe carcass bruises	8.57 ^a^	48.89 ^b^	46.67 ^bd^	84.00 ^c^	37.50 ^b^	30.62 ^b^	16.67 ^abd^	51.73 ^bd^	44.08, 7	<0.0001
Carcass bruise type										
Handling-type (%)	5.71 ^a^	51.11 ^b^	53.33 ^b^	80.00 ^c^	29.17 ^b^	28.57 ^b^	27.78 ^b^	31.03 ^b^	42.74, 7	<0.0001
Fighting-type (%)	8.57 ^a^	9.09 ^a^	6.67 ^a^	44.00 ^b^	37.50 ^b^	18.37 ^abc^	27.78 ^ab^	17.24 ^abc^	22.82, 7	0.0018
Mounting-type (%)	20.00	37.78	26.67	40.00	20.00	20.41	38.89	31.03	9.086, 7	0.2465

Abbreviations: high loading density—space allowance in the lorry within or slightly lower than recommended density of 235 kg/m^2^; low loading density—the space allowance in the lorry higher than 235 kg/m^2^; NN pigs—stress-resistant; Nn pigs—stress-carrier; healthy pigs—slaughtered pigs without clinical signs of disease at antemortem examination and pathological lesions in lungs, liver and heart at postmortem examination; diseased pigs—slaughtered pigs without clinical signs of disease at antemortem examination but with any sign of pathological lesions in lungs, liver and heart at postmortem examination; RM—foreleg angle rigor mortis; SEM—pooled standard error of means; df—degrees of freedom. Note: different letters in the same row indicate a significant difference at *p* < 0.05 ^(a–d)^.

**Table 8:** Differences in pathological lesions in slaughter pigs in relation of transportation time, RYR-1 genotype and health status (*n =* 240).

Transportation Time	Short				Long						
RYR-1 Genotype	NN		Nn		NN		Nn				
Health Status	Healthy	Diseased	Healthy	Diseased	Healthy	Diseased	Healthy	Diseased	Chi-Square	df	*p*-Value
Number of pigs	27	50	18	25	32	44	15	29			
Type of pathological lesions											
Lung lesions (%)	0.00 ^a^	82.00 ^b^	0.00 ^a^	100.00 ^c^	0.00 ^a^	84.09 ^b^	0.00 ^a^	96.55 ^bc^	182.6	7	<0.0001
Liver milk spots (%)	0.00 ^a^	66.00 ^b^	0.00 ^a^	92.00 ^c^	0.00 ^a^	45.45 ^b^	0.00 ^a^	51.72 ^b^	107.4	7	<0.0001
Pericarditis (%)	0.00 ^a^	12.00 ^a^	0.00 ^a^	36.00 ^b^	0.00 ^a^	11.36 ^a^	0.00 ^a^	10.34 ^a^	30.40	7	<0.0001
Spread of pathological lesions											
Lesions in one organ system (%)	0.00 ^a^	52.00 ^b^	0.00 ^a^	24.00 ^c^	0.00 ^a^	65.91 ^b^	0.00 ^a^	44.83 ^bc^	80.11	7	<0.0001
Lesions in two organ systems (%)	0.00 ^a^	40.00 ^b^	0.00 ^a^	48.00 ^b^	0.00 ^a^	27.27 ^b^	0.00 ^a^	48.27 ^b^	53.34	7	<0.0001
Lesions in three organ systems (%)	0.00 ^a^	8.00 ^a^	0.00 ^a^	28.00 ^b^	0.00 ^a^	6.82 ^a^	0.00 ^a^	6.90 ^a^	25.00	7	0.0008

Abbreviations: short transportation—slaughter pigs transported less than 30 min; long transportation—slaughter pigs transported more than 3.5 h; NN pigs—stress-resistant; Nn pigs—stress-carrier; healthy pigs—slaughtered pigs without clinical signs of disease at antemortem examination and pathological lesions in lungs, liver and heart at postmortem examination; diseased pigs—slaughtered pigs without clinical signs of disease at antemortem examination but with any sign of pathological lesions in lungs, liver and heart at postmortem examination; lung lesions—slaughtered pigs with any sign of pneumonia and pleurisy; liver milk spots—slaughtered pigs with at least one milk spot lesion in liver; pericarditis—slaughtered pigs with adhesion between the heart and the pericardium; df—degrees of freedom. Note: different letters in the same row indicate a significant difference at *p* < 0.05 ^(a–c)^.

**Table 9:** Influence of transportation time on the physiometabolic blood profile of slaughter pigs with different health status and RYR-1 genotype (*n =* 240).

Transportation Time (TT)	Short				Long									
RYR-1 Genotype (G)	NN		Nn		NN		Nn				Main Effects		Interaction	
Health Status (HS)	Healthy	Diseased	Healthy	Diseased	Healthy	Diseased	Healthy	Diseased	SEM	Reference Values	TT	G	HS	TT × G × HS
Number of pigs	27	50	18	25	32	44	15	29		[43]	*p*-Value			
Stress metabolites														
Lactate (mmol/L)	8.10 ^a^	12.80 ^b^	19.87 ^c^	23.30 ^d^	10.57 ^e^	15.69 ^f^	12.79 ^b^	17.16 ^g^	1.05	0.50–5.50	<0.0001	<0.0001	<0.0001	<0.0001
Glucose (mmol/L)	6.61 ^a^	8.18 ^b^	9.31 ^c^	14.90 ^d^	6.26 ^e^	7.97 ^b^	7.35 ^b^	8.49 ^bc^	0.55	3.70–6.40	<0.0001	<0.0001	<0.0001	0.0002
Stress hormones														
Cortisol (nmol/L)	265.58	291.51	251.25	256.00	268.22	244.45	286.47	277.43	38.01	76.00–88.00	0.8964	0.9886	0.9818	0.7030
ACTH (pmol/L)	1.09	0.85	1.23	1.55	0.40	0.65	0.31	0.27	0.48	-	0.0527	0.7543	0.8101	0.4870
Electrolytes														
Sodium (mmol/L)	135.63 ^a^	130.71 ^b^	125.40 ^c^	117.33 ^d^	132.23 ^b^	126.09 ^c^	130.22 ^b^	127.07 ^c^	1.45	140.00–150.00	0.0922	<0.0001	<0.0001	<0.0001
Chloride (mmol/L)	91.32 ^a^	84.78 ^b^	83.56 ^b^	79.47 ^c^	86.63 ^b^	85.37 ^b^	85.50 ^b^	84.36 ^b^	1.05	94.00–103.00	0.3110	<0.0001	<0.0001	0.0432
Stress enzymes														
CK (units/L)	1810.47 ^a^	2629.09 ^b^	3344.47 ^c^	4263.12 ^d^	3041.78 ^c^	3654.30 ^d^	3118.75 ^c^	3721.25 ^d^	211.31	66.00–489.00	0.0161	<0.0001	<0.0001	0.0010
LDH (units/L)	899.10 ^a^	1245.54 ^b^	3463.42 ^c^	4497.21 ^d^	1595.47 ^e^	2446.67 ^f^	1313.33 ^b^	2139.09 ^f^	138.04	380.00–630.00	<0.0001	<0.0001	<0.0001	0.0484
AST (units/L)	42.58 ^a^	40.17 ^a^	41.50 ^a^	86.73 ^b^	26.38 ^c^	33.76 ^d^	36.62 ^d^	34.36 ^d^	8.52	32.00–84.00	0.0010	0.0186	0.0444	0.0170
ALT (units/L)	41.67 ^a^	40.09 ^a^	42.80 ^a^	58.13 ^b^	30.83 ^c^	32.60 ^c^	34.13 ^cd^	37.53 ^d^	2.01	31.00–58.00	<0.0001	<0.0001	0.0011	0.0081
Acute-phase proteins														
Haptoglobin (mg/L)	0.25	0.25	0.28	0.29	0.26	0.26	0.19	0.23	0.04	20.00–3000.00	0.0559	0.9922	0.8369	0.6930
CRP (mg/L)	1.06	1.00	1.00	1.17	1.00	1.00	1.00	1.12	0.08	5.00–30.00	0.5665	0.2444	0.2444	0.8670
Albumin (g/L)	39.56 ^a^	33.14 ^b^	31.06 ^b^	25.96 ^c^	37.71 ^a^	34.18 ^ab^	32.58 ^b^	35.67 ^ab^	0.78	30.00–40.00	<0.0001	<0.0001	<0.0001	0.0090
Ceruloplasmin (mg/dL)	15.19 ^a^	24.49 ^b^	33.54 ^c^	45.24 ^d^	18.13 ^e^	22.60 ^b^	21.55 ^b^	25.43 ^b^	2.20	-	<0.0001	<0.0001	<0.0001	<0.0001
PON-1 (units/L)	1570.08 ^a^	966.92 ^b^	775.19 ^c^	236.79 ^d^	1414.48 ^a^	816.86 ^bc^	1031.19 ^b^	939.85 ^b^	126.39	-	0.0046	<0.0001	<0.0001	0.0500
Oxidative stress biomarkers														
MDA (nmol/mL)	3.04 ^a^	3.80 ^a^	5.31 ^b^	8.73 ^c^	4.34 ^b^	5.22 ^b^	5.33 ^b^	5.77 ^b^	0.34	-	0.7426	<0.0001	<0.0001	<0.0001
GSH (µM/L)	2.12 ^a^	1.38 ^b^	0.97 ^c^	0.46 ^d^	2.17 ^a^	1.13 ^b^	0.98 ^c^	1.02 ^c^	0.23	-	0.3426	<0.0001	<0.0001	0.0340

Abbreviations: short transportation—slaughter pigs transported less than 30 min; long transportation—slaughter pigs transported more than 3.5 h; NN pigs—stress-resistant; Nn pigs—stress-carrier; healthy pigs—slaughtered pigs without clinical signs of disease at antemortem examination and pathological lesions in lungs, liver and heart at postmortem examination; diseased pigs—slaughtered pigs without clinical signs of disease at antemortem examination but with any sign of pathological lesions in lungs, liver and heart at postmortem examination; ACTH—adrenocorticotropic hormone; CK—creatine kinase; LDH—lactic dehydrogenase; AST—aspartate amino transferase; ALT—alanine amino transferase; CRP—C-reactive protein; MDA—Malondialdehyde; GSH—Glutathione; PON-1—paraoxonase-1; SEM—pooled standard error of means. Note: different letters in the same row indicate a significant difference at *p* < 0.05 ^(a–g)^.

**Table 10:** Influence of transportation time on the rigor mortis and carcass bruises of slaughter pigs with different health status and RYR-1 genotype (*n =* 240).

Transportation Time	Short				Long				SEM	*p*-Value
RYR-1 Genotype	NN		Nn		NN		Nn			
Health Status	Healthy	Diseased	Healthy	Diseased	Healthy	Diseased	Healthy	Diseased		
Number of pigs	27	50	18	25	32	44	15	29		
Rigor mortis										
RM (°)	120.04 ^a^	117.66 ^b^	117.33 ^b^	110.40 ^c^	120.16 ^a^	115.95 ^d^	114.73 ^d^	116.59 ^bd^	0.84	<0.0001
Rigor score	1.85	1.86	1.50	1.72	1.66	1.57	1.47	1.62	0.25	0.940
Carcass bruise severity									Chi-square, df	
No carcass bruises	77.78 ^a^	26.00 ^b^	27.78 ^b^	8.00 ^b^	40.63 ^bc^	29.55 ^b^	13.33 ^bc^	31.03 ^bc^	36.90, 7	<0.0001
Moderate carcass bruises	11.11 ^a^	44.00 ^b^	33.33 ^ab^	8.00 ^a^	31.25 ^ab^	20.45 ^ab^	66.67 ^b^	17.24 ^a^	29.64, 7	0.0001
Severe carcass bruises	11.11 ^a^	30.00 ^ae^	38.89 ^ae^	84.00 ^b^	28.12 ^a^	50.00 ^ce^	20.00 ^a^	51.73 ^ce^	39.65, 7	<0.0001
Carcass bruise type										
Handling-type (%)	0.00 ^a^	10.00 ^a^	44.44 ^b^	80.00 ^c^	28.13 ^b^	36.36 ^b^	33.33 ^b^	31.03 ^b^	53.64, 7	<0.0001
Fighting-type (%)	11.11 ^a^	14.00 ^a^	5.56 ^a^	44.00 ^b^	28.13 ^b^	18.18 ^abc^	33.33 ^b^	13.79 ^a^	17.79, 7	0.0129
Mounting-type (%)	11.11	28.00	22.22	40.00	25.00	29.55	46.67	34.48	9.083, 7	0.2468

Abbreviations: short transportation—slaughter pigs transported less than 30 min; long transportation—slaughter pigs transported more than 3.5 h; NN pigs—stress-resistant; Nn pigs—stress-carrier; healthy pigs—slaughtered pigs without clinical signs of disease at antemortem examination and pathological lesions in lungs, liver and heart at postmortem examination; diseased pigs—slaughtered pigs without clinical signs of disease at antemortem examination but with any sign of pathological lesions in lungs, liver and heart at postmortem examination; RM—foreleg angle rigor mortis; SEM—pooled standard error of means; df—degrees of freedom. Note: different letters in the same row indicate a significant difference at *p* < 0.05 ^(a–e)^.

## Data Availability

The data that support the findings of this study are available from the corresponding author upon reasonable request.
